# Effects of curing concentration and drying time on flavor and microorganisms in dry salted Spanish mackerel

**DOI:** 10.1016/j.fochx.2024.101126

**Published:** 2024-01-06

**Authors:** Caiyan Jiang, Yang Liu, Wengang Jin, Kaiyue Zhu, Xiaoqing Miao, Xiuping Dong, Pengfei Jiang

**Affiliations:** a National Engineering Research Center of Seafood, School of Food Science and Technology, Dalian Polytechnic University, Dalian 116034, China; b School of Biological Science and Engineering Shaanxi Key Laboratory of Bioresources, Shaanxi University of Technology, Hanzhong 723001, China

**Keywords:** Spanish mackerel, Curing and drying, Volatile flavor compounds, Gas chromatography-ion mobility spectrometry (GC-IMS), High-throughput sequencing (HTS)

## Abstract

This study investigated the quality changes of dry salted mackerel during curing and drying process and the relationship between flavor substances and microorganisms. The results showed that the thiobarbituric acid reactive substances (TBARS) values increased gradually with the increase of salt concentration and treatment time. The total volatile base nitrogen (TVB-N) values and total viable counts (TVC) values showed the same trend. Under 3% condition, the TVB-N values exceeded the standard and was not suitable for consumption. A total of 61 volatile flavor substances were identified by Gas chromatography-ion mobility spectrometry (GC-IMS), among which aldehydes contributed the most. *Staphylococcus* and *Cobetia* were the most abundant by High-throughput sequencing (HTS). There was significant correlation between TOP15 microorganisms and TOP20 flavor substances. *Staphylococcus* and *Cobetia* were positively correlated with 13 volatile flavor substances, which contributed to the formation of flavor in naturally fermented Spanish mackerel.

## Highlights

• Spanish mackerel can reach the edible standard under appropriate conditions.
• Curing and drying preserved the flavor in Spanish mackerel.
• 51 volatile flavor substances were detected in dry salted Spanish mackerel.
• There was significant correlation between flavor substances and microorganism.

## 1 Introduction

Spanish mackerel （*Scomberomorus niphonius*） was well-liked by consumers, as an important economic fish, due to its high protein content, rich nutritional value, firm meat, and delicious taste, which was widely distributed in South Korea, Japan, China, and other regions, and in China mainly distributed in the East China Sea, Yellow Sea, and the Bohai Sea (Huang et al., 2019). The fishing of Spanish mackerel is seasonal, so choosing appropriate intensive processing methods can not only retain the rich nutrients but also prolong the preservation period of Spanish mackerel. Salting and drying are traditional methods that were widely used in coastal areas for the further processing of aquatic products. During processing, proteins and lipids under the action of microorganisms and endogenous enzymes give aquatic products a unique flavor, which is favored by the public (Wang et al., 2022). As a fish preservation method with a long history, pickled fish is often processed in small workshops, the processing method is single, and most of them are empirical processing, which can’t guarantee the stability of the products. Adel, Awad, Mohamed and Mohamed (2021) evaluated the free fatty acids, volatile base nitrogen, trinitrobenzene sulfonic acid, peroxide value, thiobarbituric acid value, and microbiological analysis of five different dry salted fish products. The results showed that there were great differences in chemical composition and physicochemical indicators of dry salted fish in different areas, indicating that different producing areas and processing techniques had a great influence on dry salted fish. In summary, the production conditions and technology of salted fish have a great impact on its quality, which provides theoretical support for the industrial production of salted fish.

Food flavor mainly refers to the comprehensive perception of flavor substances in people's senses, and it is one of the important indicators for people to evaluate food quality. With the gradual progress of science and technology, more and more detection techniques have been applied to the detection of food flavor. At present, there are mainly electronic nose technology, headspace analysis technology, gas chromatography–olfactometry, gas–liquid chromatography technology, gas chromatography-mass spectrometry technology, and gas chromatography-ion mobility spectrometry (Arroyo-Manzanares et al., 2019).

Gas chromatography-ion mobility spectrometry (GC-IMS) is a technique which rose in recent years with the advantages of high sensitivity, fast response time, ease of operation, and relatively low cost. Chai et al. (2023) used different detection techniques to analyze star anise essential oil extracted by different extraction methods. The results showed that GC-IMS technology can analyze volatile flavor compounds in samples more quickly and accurately than electronic nose and gas chromatography-mass spectrometry. GC-IMS technology has been extensively applied to differentiate samples of different origins, species, and treatments (Li et al., 2022, Li et al., 2022, Li et al., 2022, Yu et al., 2021). And has covered food, industry, medicine, environmental testing, and other fields (Li et al., 2022, Li et al., 2022, Li et al., 2022). Therefore, the GC-IMS technique is a fast and accurate method for the detection of flavor substances in samples.

High-throughput sequencing (HTS) is an analysis technique based on DNA fragments to obtain effective sequence information. HTS can accurately and quickly identify a large amount of microbiological information in samples. In recent years, it has been widely used in the food industry and has become one of the main methods to study microbial diversity (Huang, Peng, Chen, Yan & Zhang, 2023). Kobayashi et al. (2016) studied fermentation-related microorganisms in Myanmar salted fish, providing data reference for clarifying their effects on the salting and ripening of salted fish. Some microorganisms (*L. plantarum X23*) play an important role in the preservation of salted fish, which can significantly reduce the content of histamine and putrescine in salted fish (Feng, Li, Tuo, & Ma, 2021). The composition and changes of dominant microflora in each link of the tangyuan production chain (Suo, et al., 2023), in pasteurized milk during storage (Ding, et al., 2023), and in modified atmosphere packaging mussel samples during storage at 4 °C (Olumide, Christopher, Christopher & Roger, 2019) were analyzed by HTS. The change law of dominant microflora was explored to provide a theoretical basis for inhibiting the growth of rotten bacteria and controlling the quality of products.

Microorganisms are important in the formation of fermented food flavor, in which the metabolites such as amino acids, active metabolites, and organic acids in the fermentation process will change the flavor of food and improve the quality of product. Some researchers have made a comprehensive study on the relationship between flavor substances and microorganisms in industrial kimchi brine, which shows that microorganisms are positively correlated with esters, alcohols, and sulfides, indicating that there is a close relationship between microorganisms and flavor substances (Zhao, Suyama, Wu & Zhang, 2022). Liu, Wang, Sun and Li (2020) used HS-SPME-GC/MS, HST, and ITS sequencing techniques to detect volatile flavor compounds and microbial sequences during the fermentation of traditional fermented glutinous rice wine, and considered that microorganisms played a major role in the formation of flavor, which provided data support for further exploration of the formation mechanism of flavor substances in rice wine.

In this study, the changes in safety indicators (TBARS, TVB-N, and TVC) of dry salted Spanish mackerel during curing and drying were analyzed. The volatile flavor compounds produced by dry salted Spanish mackerel were detected by GC-IMS. Meanwhile, the changes in bacterial community and dominant bacteria in the samples were analyzed by high throughput sequencing analysis. This research objective is to explore the relationship between volatile flavor substances and bacterial community in dry salted Spanish mackerel and to provide a theoretical basis for its industrial production.

## 2 Materials and methods

### 2.1 Sample preparation

**Fig. 1:**
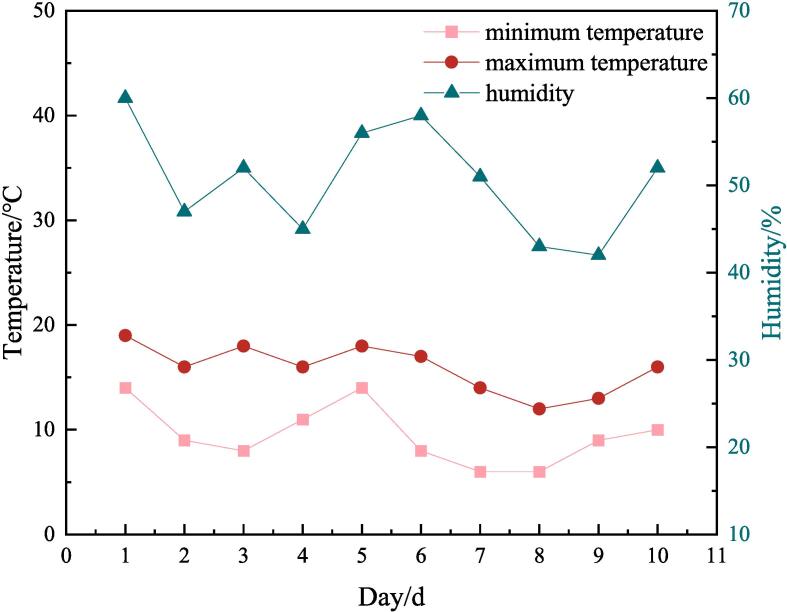
Curve of temperature and humidity variation during curing and drying.

Fresh Spanish mackerels were captured from Changhai County (Dalian City, Liaoning Province, China) in October 2022. Samples of moderate size, length (40 ± 5) cm, and weight (400 ± 50) g were selected for pretreatment. Briefly, Spanish mackerel was cut open along the spine, the internal organs were removed, and the blood stains were cleaned. The pretreated Spanish mackerel were pickled by dry curing method, the salt of 3 %, 6 %, and 9 % of the fish body weight was evenly applied to the surface of the fish body, and then curing for 48 h, subsequently, the fish were hung head up to dry for 8 days, the above operations were in a cool and ventilated place in late October 2022, then dried Spanish mackerel was obtained after these processes. The change curve of temperature and humidity was shown in Fig. 1. The dried Spanish mackerels were divided into 19 groups according to salt concentration and treatment time, which were BF00, fresh samples; BM30, 3 % curing 24 h; BA30, 3 % curing 48 h; BA32, 3 % drying 2 d; BA34, 3 % drying 4 d; BA36, 3 % drying 6 d; BA38, 3 % drying 8 d; BM60, 6 % curing 24 h; BA60, 6 % curing 48 h BA62, 6 % drying 2 d; BA64, 6 % drying 4 d; BA66, 6 % drying 6 d; BA68, 6 % drying 8 d; BM90, 9 % curing 24 h, BA90, 9 % curing 48 h, BA92, 9 % drying 2 d, BA94, 9 % drying 4 d, BA96, 9 % drying 6 d, BA98, 9 % drying 8 d and then stored at −80 °C for subsequent analyses.

### 2.2 Safety indicators

#### 2.2.1 Thiobarbituric acid reactants (TBARS)

The values of TBARS were determined by referring to the method of Liza et al. (2005) with a slight modification. The Spanish mackerel samples (1.0 ± 0.1 g) were fully ground and added to the 5 mL mixture (0.375 % TBA, 15 % TCA, 0.25 mol/L HCL) and then swirled and oscillated. After 10 min in boiling water, until the solution turned pink and cooled in running water, then centrifugation (CR22N high-speed refrigerated centrifuge, Koki Holdings Co., Ltd, Ibaraki, Japan) at 4 °C at 8000 r/min for 10 min. The supernatant (200 μL) was taken in 96-well plates and its absorbance values were determined under 532 nm. The content of TBARS was calculated according to the formula as follows:

$$\text{TBARS}=\text{Sample}\;A_{532}\times 2.77$$

#### 2.2.2 Total volatile basic nitrogen (TVB-N)

TVB-N was measured by the Kjeldahl method (Ling et al., 2022). Samples (10 ± 0.001 g) were added into 75 mL distilled water, then homogenized with a homogenizer (clapping stomacher SH-400A, Shanghai Hegong Scientific Instrument Co., Ltd. China). The sample was fully exposed to the solution and then stood at room temperature for 30 min. The automatic Kjeldahl nitrogen analyzer was used for analysis and determination. The calculation formula is as follows:

$$X=(V_{1}-V_{2})\times c\times 14/m\times 100$$

Where *X* was the TVB-N value (mg/100 g), *V_1_* and *V_2_* were the volume of hydrochloric acid standard titration solution consumed by the test solution and blank, *c* was the concentration of the standard titration solution of hydrochloric acid, 14 was the mass of nitrogen equivalent to titrate the standard solution of 1.0 mL hydrochloric acid (1.000 mol/L), *m* was the mass of sample, 100 was the conversion factor.

#### 2.2.3 Total viable counts (TVC)

The sample (25 g) was homogenized in sterile saline (225 mL) and homogenized for 1 min in a homogenizer (clapping stomacher SH-400A, Shanghai Hegong Scientific Instrument Co., Ltd. China) to make a 1:10 mixture. The bacterial suspension (1 mL) diluted 2–3 times was put into an aseptic petri dish and spread evenly on plate count agar. The plates were inverted in a 30 °C incubator for 72 h and then counted (Ling et al., 2022).

### 2.3 Gas chromatography-ion mobility spectrometry (GC-IMS) analysis

The volatiles in dry salted Spanish mackerels were identified by GC-IMS (FlavourSpec®, G.A.S. Instrument, Shandong Haineng Scientific Instrument Co., Ltd, China). Briefly, samples (2 g) were hatched in a 20 mL headspace bottle for 20 min at 40 °C with an incubation speed of 500 r/min. According to Jiang et al. (2022) with a slight modification. Then 500 μL of headspace gas was automatically injected into the injector at 85 °C with splitless mode, nitrogen (99.99 %) was used as carrier gas and drift gas, and instrument cleaning time was 0.5 min. The flow rate started with 2 mL/min for 2 min, increased to 10 mL/min over 8 min, then flow ramped up to 100 mL/min at 10 min, and then increased to 150 mL/min within 15 min. Meanwhile, the drift gas was set to 150 mL/min. The total GC runtime was 25 min. The chromatographic separation was equipped with an MXT-5 capillary column (15 m × 0.53 mm, 1 μm film thickness), which temperature kept at 60 °C, and the drift tube temperature kept at 45 °C. Each sample was measured thrice.

### 2.4 Relative odor activity value (ROAV)

The calculation of ROAV in dry salted Spanish mackerel was referenced from Jiang et al. (2022). The contribution of different volatile flavor compounds in dry salted Spanish mackerel to the overall flavor was evaluated, and a new parameter was defined to determine the main flavor substances. The flavor compounds that contribute most to the sample are defined as ROAV_stan_ = 100 and the ROAV values of other volatile flavor compounds were calculated as follows:

$$\mathrm{ROA}V_{A}=C_{A}/C_{\mathrm{stan}}\times T_{\mathrm{stan}}/T_{A}\times 100\%.$$

In this formula, T_stan_ means the threshold corresponding to the greatest contribution to volatile flavor compounds, T_A_ means the threshold corresponding to each volatile flavor compound, C_stan_ means the relative content of the most volatile flavor compounds, and C_A_ means the relative content of each volatile flavor compound.

### 2.5 Microbial community analysis

The total DNA from dry salted Spanish mackerel was extracted by the CTAB method, and the concentration and purity of DNA extraction were detected by agarose gel electrophoresis. The extracted genome was used as the amplification template, and the V3-V4 variable region was amplified using two primers 341F (5′-CCTACGGGNGGCWGCAG-3′) and 805R (5′-GACTACHVGGGTATCTAATCC-3′). PCR reactions were done according to Jiang et al. (2022).

The PCR products were detected by 2 % agarose gels, and the products were recovered by AMPure XT beads kit. The purified PCR products were evaluated by Agilent 2100 biological analyzer (Agilent, USA) and Illumina (Kapa Biosciences, Woburn, MA, USA) quantitative kit, and the qualified concentration should be above 2 nM. After gradient dilution of the qualified sequencing, the libraries were mixed according to the required sequencing quantity and denatured into single strands by NaOH to be sequenced on the computer. 2 × 250 bp double-terminal sequencing was carried out with NovaSeq 6000 sequencer, and the corresponding reagent was NovaSeq 6000 SP Reagent Kit (500 cycles). Through qiime dada2 denoise-paired calling DADA2 for length filtering and denoising, the ASV (feature) feature sequence was obtained. According to ASV (feature) sequence files, SILVA (Release 138, https://www.arb silva.de/documentation/release138/) database was used to annotate species.

### 2.6 Statistical analysis

Statistical data were analyzed using SPSS 26.0 (IBM, Inc., Armonk, NY, USA), and one-way analysis of variance (ANOVA) was performed using Duncan's test (*P* < 0.05) to determine the difference between different treatments. The diagrams were obtained using Origin 9.0. The GC-IMS data were analyzed by Laboratory Analytical Viewer, Reporter, Gallery Plot, and Dynamic PCA plug-ins. The volatile flavor compounds in the samples were qualitatively analyzed by comparing them with the built-in database of the instrument (GC × IMS Library Search NIST database and IMS database). The correlation was determined by Pearson correlation analysis. All experimental results were repeated three times, and the results were expressed as the mean values ± standard errors.

## 3 Results and discussion

### 3.1 Results of safety indicators

#### 3.1.1 TBARS analysis

**Fig. 2:**
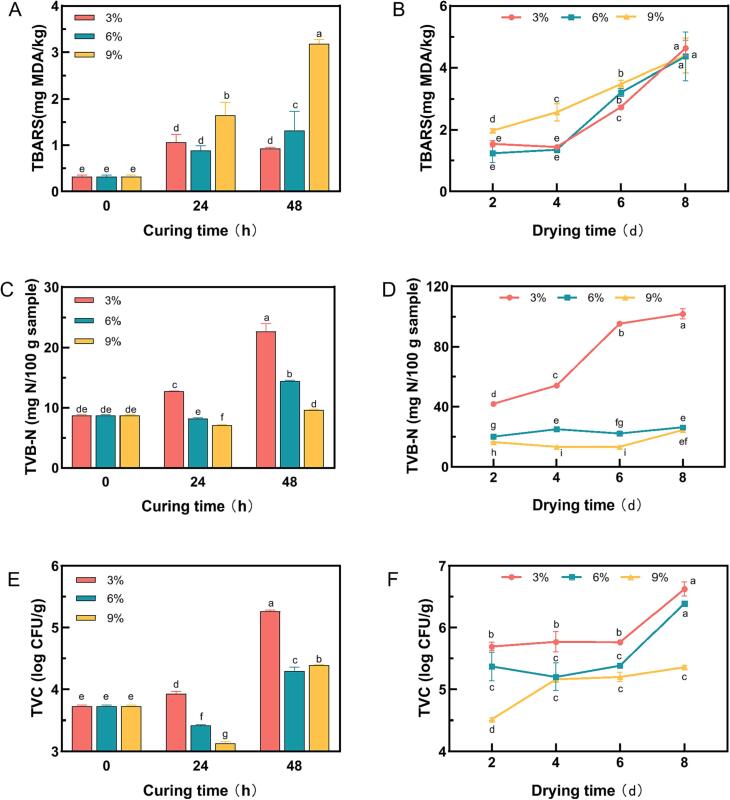
Changes in TBARS (A, B), TVB-N (C, D) and TVC (E, F) of dry salted Spanish mackerel during different curing time and drying time respectively. Values with different letters are significantly different (p < 0.05).

As an important factor in evaluating the quality of meat products, the flavor was formed by a series of reactions of precursor substances. As a flavor precursor, lipid plays a vital role, but excessive fat oxidation will affect the flavor of meat products, meanwhile, pose a threat to human health (Adam, Monika, Katarzyna, Renata & Janusz, 2020). Changes in TBARS of dry salted Spanish mackerel were shown in Fig. 2A and B. With the extension of curing and drying time, the TBARS value in dry salted Spanish mackerel gradually increased. Under the condition of 9 % salt concentration, the TBARS value was significantly higher than that of other concentrations, the result suggests that salt could promote lipid oxidation. Min, Cordray and Ahn (2010) reported that sodium chloride can be used as an oxidant, which may be because sodium chloride destroys the integrity of the cell membrane and releases free iron from binding macromolecules, and the presence of sodium chloride reduces the activity of antioxidant enzymes, thus promoting fat oxidation.

#### 3.1.2 TVB-N analysis

The degree of protein decomposition in Spanish mackerel samples can be expressed by the value of TVB-N, as shown in Fig. 2C and D. Mohdaly, Mahmoud, Ramadan and Roby (2021) reported that the higher TVB-N value may be due to the decomposition of proteins into volatile nitrogen compounds under the combined action of microorganisms and enzymes. The TVB-N value of fresh Spanish mackerel was 8.74 mg/100 g. With the extension of drying time, the TVB-N value of dry salted Spanish mackerel showed an overall upward trend, in which the values of 2–4 d and 4–6 d (6 % and 9 %) TVB-N decreased slightly, which may be due to the diversity of samples (Lv, Huang, Aheto, Mu & Tian, 2018). The results showed that higher salt concentration could inhibit the growth of endogenous enzymes and microorganisms in Spanish mackerel meat, slow down the rate of protein decomposition, and the deterioration of fish meat (Fan, Luo, Yin, Bao & Feng, 2014). Furthermore, it is obvious that the TVB-N value of dry salted Spanish mackerel with 3 % salt concentration was the highest, which was significantly higher than that of other concentrations, and on the second day of drying, the TVB-N value reached 41.82 mg/100 g. The maximum recommended limit set of TVBN was not exceed 30 mg/100 g (Bekhit, Holman, Giteru, & Hopkins, 2021). That indicating dry salted Spanish mackerel under this treatment condition had spoiled and were not suitable for consumption. Under the curing condition of 6 % salted concentration, which TVB-N value was lower than the national standard limit, slightly higher than 9 % salting concentration.

#### 3.1.3 TVC analysis

The change of the TVC of microorganisms from dry salted Spanish mackerel under different curing and drying conditions was shown in Fig. 2E and F. The initial bacterial counts in Spanish mackerel samples were 3.73 log CFU/g, proving that the initial quality of the fish was excellent. After curing for 24 h at different salt concentrations, under the condition of 3 % salt concentration, the TVC increased slightly but decreased significantly under 6 % and 9 %. The TVC values increased sharply after 48 h of curing under three different salt concentrations, which may be because the higher salt concentration at the initial stage of pickling inhibited the growth of microorganisms (Seyed, Masoud, Seyed, & Seyed, 2009). And with time, most microorganisms adapted to the living environment, meanwhile, the nutrients were sufficient and suitable for microbial growth, resulting in a sharp increase in their content. In the late drying stage, the TVC increased at a relatively slow rate, which probably owing to the accumulation of lactic acid in the growth process, thus inhibiting the growth of microorganisms (Lv, Yin, Wang, Che & Kong, 2021). The higher the salt concentration, the smaller the TVC value. When curing at 9 % concentration, TVC values were significantly lower than the other two groups. While at 3 %, TVC values were significantly higher than 6 % and 9 %. The TVC values in the samples reached the maximum on the 8th day under different salting concentrations, which were 6.63 log CFU/g, 6.39 log CFU/g, and 5.36 log CFU/g, respectively. Indicating that NaCl can enormously inhibit the growth of microorganisms, which was because the hypertonic environment caused by pickling caused cytoplasmic water loss and the synthesis of biomacromolecules was inhibited, which was not suitable for the growth of microorganisms (Oujifard, Benjakul, Nirmal, & Bashirzadeh, 2021).

### 3.2 Volatile compounds identified in Spanish mackerel by GC-IMS

#### 3.2.1 Analysis of flavor composition spectrum

**Fig. 3:**
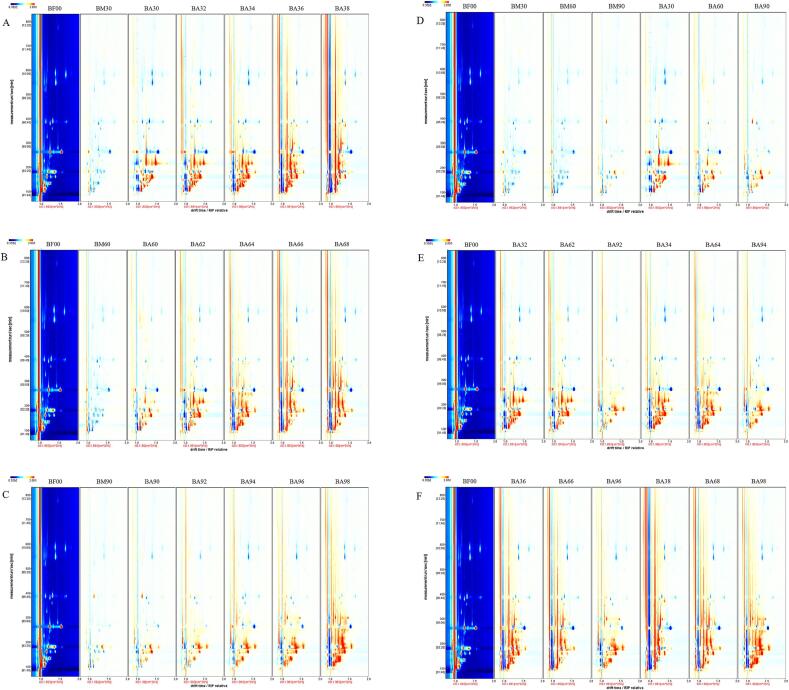
Comparison of volatile flavor substances in Spanish mackerel with different treatment methods and time: (A) 3 % curing 48 h and drying 8 d; (B) 6 % curing 48 h and drying 8 d; (C) 9 % curing 48 h and drying 8 d; (D) 3 %, 6 %, and 9 % curing 24 h and 48 h; (E) 3 %, 6 %, and 9 % curing 2 d and 4 d; (F) 3 %, 6 %, and 9 % curing 6 d and 8 d.

As shown in Fig. 3, different treatment methods and times had great effects on the flavor substances in Spanish mackerel. The abscissa represented the ion migration time and the ordinate represented the gas chromatographic retention time. For the sake of observation, taking the fresh Spanish mackerel sample (BF00) as a reference, the other samples deducted the same part as BF00, the background was white, red indicated that the content of the substance was higher than BF00, the darker the red was, the more the content of the substance was, the blue indicated that the content of the substance was lower than that of BF00, and the darker the blue was, the less the substance was. Fig. 3-A, B, and C demonstrated that the volatile flavor substances in mackerel gradually increased with the extension of time when the salt concentration was 3 %, 6 %, and 9 % respectively. It reached its maximum on the 8th day. Fig. 3-D, E, and F demonstrated the comparison of flavor substances in pickled mackerel with different salt concentrations at the same time point. The graph showed that at the same time point, the samples contained the most flavor substances at 3 % salt concentration. Ammonia and trimethylamine increased gradually with drying time increasing and were significantly higher in 3 % than in other salt concentrations. Trimethylamine was the main component of spoilage taste in aquatic products and its value was usually used as an important index to evaluate the quality and shelf life of aquatic products (Judite, Pedro, & Emílio, 2004). But above all, it is clear that samples cured at 3 % salt concentration and left to dry for 6 and 8 d were no longer suitable for consumption. It can be intuitively seen from Fig. 3 that there were certain differences between volatile flavor substances under different treatment conditions, but it was difficult to determine the changes of single compound, and the changes of single compound were detailed in the fingerprint, like Liu et al. (2021).

#### 3.2.2 Qualitative analysis of flavor components

**Fig. 4:**
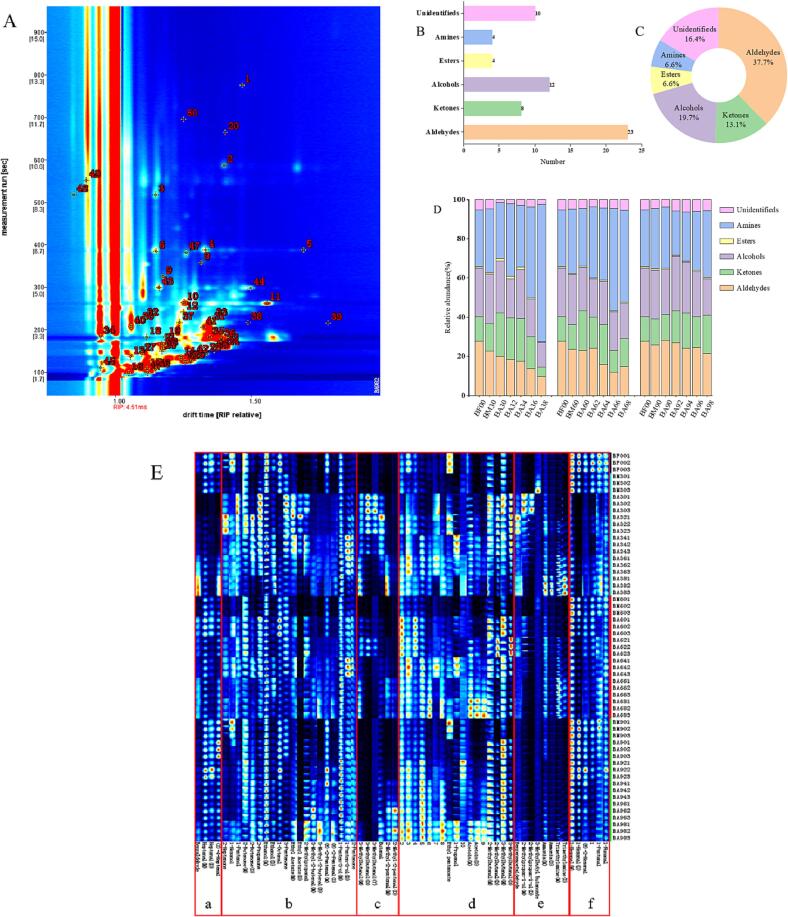
Characteristics of volatile flavor compounds in dry salted Spanish mackerel under different curing and drying time, qualitative analysis of volatile compounds (A), number and percentage of volatile compounds (B) and percentage (C), proportion of volatile flavor compounds (D) and fingerprint (E).

The volatile flavor compounds in dry salted Spanish mackerel were qualitatively analyzed by using the VOCal software of the instrument. The results of the qualitative analysis of volatile flavor compounds were shown in Fig. 4. Each number represents a volatile flavor compound, which corresponds to the compounds in Table 1. The retention time of volatile flavor compounds was compared with the data in the IMS database. A total of 61 volatile flavor compounds were identified from samples, including 23 aldehydes, 8 ketones, 12 alcohols, 4 esters, 4 amines, and 10 undetermined components (37.7 % aldehydes, 13.1 % ketones, 19.7 % alcohols, 6.6 % esters, 6.6 % amines and 16.4 % undetermined components).

**Table 1:** Volatile flavor compounds in dry salted Spanish mackerel.

Count	Compound	Odor type	CAS	Formula	MW	RI	Rt [sec]	Dt [a.u.]
1	1-Nonanal	Waxy, aldehydic, rose, fresh, orris, orange peel, fatty	C124-19-6	C_9_H_18_O	142.2	1105.2	775.718	1.4690
2	1-Octanal	Aldehydic, waxy, citrus, orange, peel, green, herbal, fresh, fatty	C124-13-0	C_8_H_16_O	128.2	1011.4	587.311	1.4024
3	Benzaldehyde	Cherry, almond, sweet, burnt sugar, sharp, strong, bitter	C100-52-7	C_7_H_6_O	106.1	974.5	517.042	1.1503
4	Heptanal	Citrus, ozone, fat, herbal, fresh, wine-lee, rancid, fatty, aldehydic, green	C111-71-7	C_7_H_14_O	114.2	898.4	387.534	1.3348
5	Heptanal (D)	–	C111-71-7	C_7_H_14_O	114.2	898.4	387.534	1.6946
6	(Z)-4-heptenal	Oily, fatty, green, dairy, milky, creamy	C6728-31-0	C_7_H_12_O	112.2	896.3	384.444	1.1521
7	2-Heptanone	Coconut, soap, herbal, sweet, woody, fruity, spicy, cinnamon	C110-43-0	C_7_H_14_O	114.2	893.3	380.077	1.2626
8	1-Hexanol	Oil, alcoholic, ethereal, resin, fusel, sweet, fruity, flower, green	C111-27-3	C_6_H_14_O	102.2	876.1	357.998	1.3178
9	(E)-2-Hexenal	Green, fatty	C6728-26-3	C_6_H_10_O	98.1	847.9	324.545	1.1780
10	1-hexanal	Fresh, green, fatty, aldehydic, grass, leafy, fruity, sweaty	C66-25-1	C_6_H_12_O	100.2	787.6	263.327	1.2555
11	1-hexanal (D)	–	C66-25-1	C_6_H_12_O	100.2	786.1	261.989	1.5608
12	1-Pentanol	Oil, balsamic, vanilla, fusel, sweet, balsam	C71-41-0	C_5_H_12_O	88.1	765.3	241.362	1.2536
13	2-Butanone	Ethereal, ether, fruity, acetone, camphor	C78-93-3	C_4_H_8_O	72.1	586.7	135.596	1.0597
14	2-Butanone (D)	–	C78-93-3	C_4_H_8_O	72.1	580.4	133.337	1.2447
15	2-Propanone	Solvent, ethereal, apple, pear	C67-64-1	C_3_H_6_O	58.1	499.0	107.385	1.1207
16	Ethanol	Alcoholic, ethereal, medical, sweet, strong	C64-17-5	C_2_H_6_O	46.1	464.7	98.032	1.0521
17	Ethanol (D)	–	C64-17-5	C_2_H_6_O	46.1	467.5	98.751	1.1223
18	3-Pentanone	Ethereal, acetone, ether	C96-22-0	C_5_H_10_O	86.1	694.5	182.032	1.1186
19	1-pentanal	Oil, balsamic, vanilla, fusel, sweet, balsam	C110-62-3	C_5_H_10_O	86.1	694.5	182.032	1.1900
20	3-methylbutyl butanoate	Fruity, green, apricot, pear, banana	C106-27-4	C_9_H_18_O_2_	158.2	1053.1	664.661	1.4067
21	2-methyl Butanal	Musty, cocoa, phenolic, coffee, nutty, malty, fermented, fatty, alcoholic	C96-17-3	C_5_H_10_O	86.1	664.2	166.64	1.1723
22	2-methyl Butanal (D)	–	C96-17-3	C_5_H_10_O	86.1	664.2	166.64	1.4023
23	3-Methyl butanal	Peach, sour, chocolate, ethereal, malt, fatty, aldehydic	C590-86-3	C_5_H_10_O	86.1	639.8	156.169	1.1803
24	3-Methyl butanal (D)	–	C590-86-3	C_5_H_10_O	86.1	641.3	156.785	1.4052
25	2-Methylpropan-1-ol	Ethereal, winey, cortex	C78-83-1	C_4_H_10_O	74.1	621.7	148.807	1.1745
26	2-Methylpropan-1-ol (D)	–	C78-83-1	C_4_H_10_O	74.1	624.0	149.706	1.3652
27	Ethyl Acetate	Pineapple, ethereal, sweet, anise, fruity, balsam, weedy, green	C141-78-6	C_4_H_8_O_2_	88.1	606.6	142.937	1.0992
28	Ethyl Acetate (D)	–	C141-78-6	C_4_H_8_O_2_	88.1	606.2	142.824	1.3379
29	2-Methylpropanal	Fresh, aldehydic, floral, green	C78-84-2	C_4_H_8_O	72.1	557.6	125.489	1.2803
30	3-methyl-2-butenal	Brown, sweet, pungent, fruity, cherry, nutty, almond	C107-86-8	C_5_H_8_O	84.1	738.1	216.541	1.0924
31	3-methyl-2-butenal (D)	–	C107-86-8	C_5_H_8_O	84.1	738.1	216.541	1.3548
32	(E)-2-Pentenal	Pungent, green, fruity, apple, orangey, tomato	C1576-87-0	C_5_H_8_O	84.1	749.0	226.202	1.1083
33	(E)-2-Pentenal (D)	–	C1576-87-0	C_5_H_8_O	84.1	748.3	225.577	1.3616
34	1-Penten-3-ol	Butter, pungent, tropical, horseradish, green, vegetable, bitter, fruity	C616-25-1	C_5_H_10_O	86.1	696.4	183.378	0.9486
35	1-Penten-3-ol (D)	–	C616-25-1	C_5_H_10_O	86.1	694.1	181.729	1.3493
36	2-Pentanone	Potato, alcohol, ether, ethereal, wine, banana, fishy, fruit, sweet, woody, fruity	C107-87-9	C_5_H_10_O	86.1	688.6	177.779	1.3921
37	3-methylbutanol	Fusel, oil, alcoholic, whiskey, fruity, banana	C123-51-3	C_5_H_12_O	88.1	737.4	216.0	1.2397
38	3-methylbutanol (D)	–	C123-51-3	C_5_H_12_O	88.1	737.4	216.0	1.4906
39	3-methylbutanol (T)	–	C123-51-3	C_5_H_12_O	88.1	736.9	215.514	1.7847
40	Acetoin	Butter, cream, milky, fatty, creamy, sweet, dairy, buttery	C513-86-0	C_4_H_8_O_2_	88.1	726.5	206.764	1.0615
41	Acetoin (D)	–	C513-86-0	C_4_H_8_O_2_	88.1	725.3	205.791	1.3260
42	Butanal	Pungent, cocoa, musty, green, malty, bready	C123-72-8	C_4_H_8_O	72.1	597.3	139.475	1.2919
43	2-Methyl-2-pentenal	Brown, sweet, pungent, fruity, cherry, nutty, almond	C623-36-9	C_6_H_10_O	98.1	824.7	299.537	1.1634
44	2-Methyl-2-pentenal (D)	–	C623-36-9	C_6_H_10_O	98.1	823.5	298.261	1.4992
45	Trimethylamine	Sweaty, fish, fishy, oily, rancid,	C75-50-3	C_3_H_9_N	59.1	508.2	110.039	0.9497
46	Trimethylamine (D)	–	C75-50-3	C_3_H_9_N	59.1	492.7	105.609	1.1519
47	Ethyl pentanoate	Yeast, apple, pineapple, sweet, fruity, tropical, green	C539-82-2	C_7_H_14_O_2_	130.2	894.6	381.992	1.2624
48	Ammonia	Ammoniacal	C7664-41-7	NH_3_	17.0	974.8	517.467	0.8497
49	Ammonia (D)	–	C7664-41-7	NH_3_	17.0	991.7	551.695	0.8960
50	benzeneacetaldehyde	Green, sweet, floral, hyacinth, clover, honey, cocoa	C122-78-1	C_8_H_8_O	120.2	1068.4	695.551	1.2546
51	1-Propanol	Alcoholic, fermented, alcohol, musty, fusel, pungent, peanut	C71-23-8	C_3_H_8_O	60.1	553.8	124.224	1.2594

MW: molecular weight, RI: retention index, Rt: retention time from GC-IMS, Dt: drift time from GC-IMS, (D) indicated dimer, (T) indicated trimer.

From the percentage accumulation (Fig. 4D), it is clear that with the extension of drying time, the content of aldehydes decreased gradually, and the decrease was larger under a 3 % salt concentration. In the meanwhile, aldehydes exerted an enormous function on the final flavor presentation of dry salted mackerel because of their low threshold (Xu, et al., 2022). With the increase of salt concentration, the formation rate of amines decreased substantially, and the contents of amines in samples cured with 9 % salt concentration were the least. Low salt conditions can produce unpleasant odors, possibly due to the rapid growth of spoilage microbe under low salt conditions, and previous studies also reported consistent results (Liao, Luo, Huang, & Xia, 2023). The changes of other volatile flavor compounds were relatively small, which were discussed in detail below.

#### 3.2.3 Gallery plots for volatile compounds

Through the Gallery Plot plug-in in the instrument, all the ion peaks were selected to draw fingerprints to compare the differences of volatile flavor compounds in samples under different treatment conditions more intuitively. As is shown in Fig. 4E, each row represented all the signal peaks contained in the samples under the same treatment conditions, and each column represented all signal peaks of the same volatile flavor substance under different treatment conditions. The darker the red was, the more the content of the substance was. Region a contained benzaldehyde, heptanal (M, D), and (Z)-4-heptenal. The contents of benzaldehyde were the highest at 3 % salt concentration for 8 days, while the contents of benzaldehyde were relatively small or even none under other conditions. The contents of the other three volatile flavor substances were relatively small at 3 % curing concentration, and the highest at 9 %.

The main volatile flavor substances contained in region c were 3-methyl butanol (M, D, T), butanal, and 2-methyl-2-pentenal (M, D). The contents of 3-methylbutanol and its dimer and trimer were the highest when cured at 3 % salt concentration for 48 h. The content of butanal reached the highest at 3 % salt concentration for 2 days, and the contents of 2-methyl-2-pentenal monomer and dimer were higher at the later stage of drying, while the content of 2-methyl-2-pentenal was relatively higher at 9 % salt concentration.

It can be seen from the fingerprint that there were fewer types of volatile flavor substances in fresh samples, mainly aldehyde (region f), including 1-nonanal, 1-pentanal, (E)-2-hexenal, 1-Hexanal (M, D). With the extension of treatment time, the content of five volatile flavor compounds gradually decreased, and with the gradual increase of salt concentration, the decreasing rate of aldehydes decreased gradually, indicating that higher salt concentration could better retain the unique flavor components contained in fresh Spanish mackerel. The content of amines (region e) (ammonia, trimethylamine) gradually increased and reached the maximum at 8 days of airing, and the peak value was the highest at 3 % salt concentration curing, which was consistent with the above results of TVB-N, illustrating that lower salt concentration can’t inhibit the action of enzymes and bacteria, which led to the decomposition of protein to produce a series of unpleasant odor substances (Liao et al., 2023). 2-Methylpropan-1-ol (M, D) and Benzeneacetaldehyde were mainly concentrated in 3 % salt concentration for 48 h and the initial stage of curing (region e), and the contents were less at other curing concentrations.

The main volatile flavor compounds in dry salted Spanish mackerel were concentrated in regions b and d, and the volatile flavor substances in region b can be detected in different curing concentrations and drying times. Including 2-heptanone, 1-hexanol, 1-pentanol, 2-butanone (M, D), acetone, ethanol (M, D), 1-octanal, 3-pentanone, ethyl acetate (M, D), 2-methyl propanal, 3-Methyl-2-butenal (M, D), (E)-2-pentenal (M, D), 1-penten-3-ol (M, D), 2-pentanone. These substances were the common volatile flavor compounds in dry salted mackerel under different salt concentrations and different drying times.

Region d contained 3-methyl butanal (M, D), 2-methyl butanal (M, D), acetoin (D), 1-propanol, ethyl pentanoate, and most of the undetermined flavor substances. The contents of 3-methyl butanal (M, D) and 2-methyl butanal (M, D) decreased gradually with the drying time at each salt concentration. To reduce the effect of a sour taste on the final taste of dry salted Spanish mackerel, the salt concentration should be reduced as much as possible. The existence of ethyl acetate, ethyl valerate and other esters can enhance the characteristic flavor of fermented food by reducing the foul odor (Lee, Lee, Choi, Hurh, & Kim, 2013). The threshold value of ketones such as 2-butanone, 2-pentanone, 3-hydroxy-2-butanone was lower than that of aldehydes, but their presence can reduce the fishy taste (Flores, 2018).

#### 3.2.4 ROAV calculations for volatile compounds in dry salted Spanish mackerel

**Fig. 5:**
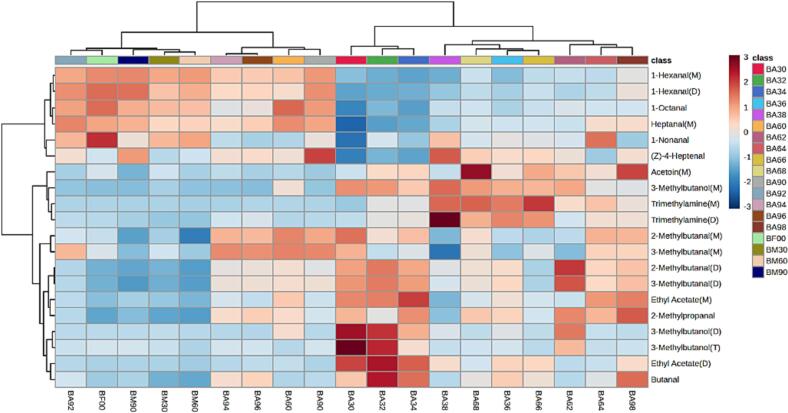
Heat map analysis of ROAV values of main volatile flavor substances in dry salted Spanish mackerel.

Many kinds of volatile flavor compounds were detected in dry salted Spanish mackerel samples, but only some of them contributed greatly to the formation of the final flavor, and the other volatile flavor compounds played a role in modifying the overall flavor. The contribution of volatile flavor compounds to the flavor characteristics of dry salted Spanish mackerel was mainly determined by its threshold and relative content. In this study, the peak volumes of 51 volatile flavor compounds detected by GC-IMS were treated by the normalization method, and the relative content of each substance was obtained, but the main flavor substances in dry salted Spanish mackerel could not be screened because the threshold of different compounds was different, and the lower the threshold was, the greater the contribution of the substance to the overall flavor. Therefore, ROAV was used to screen the volatile flavor compounds in samples under different conditions. A high ROAV value represented that it made a great contribution to the overall flavor. The substance with ROAV ≥ 1 was the key volatile component in the sample, and the substance 0.1 ≤ ROAV < 1 could modify the overall flavor of the sample (Xu et al., 2022). The threshold of (Z)-4-heptenal was the lowest among the flavor substances detected in dry salted Spanish mackerel, which was defined as the key flavor substance, ROAV_stan_ = 100. A total of 20 kinds of volatile flavor compounds with ROAV ≥ 1 was screened, the main flavor substances in dry salted Spanish mackerel were different under different conditions, so the heat maps of 20 key volatile flavor compounds were drawn as shown in Fig. 5. BA60, BA94, BA96, BA90, BA92, BM30, BM60, BF00, and BM90 were grouped into one group, in which the volatile flavor compounds were similar, including 1-hexanal (M, D), 1-octanal, heptanal (M), 1-nonanal and (Z)-4-heptenal. The volatile flavor compounds in dry salted mackerel were similar when pickled for 24 h and 9 % salt concentration, indicating that the flavor components in samples did not change significantly after pickling for 24 h, simultaneously, higher salt concentration could better maintain the original flavor substances in samples. The proportion of straight-chain aldehydes is large, indicating that fat oxidation is active in the salting process, and plays an important role in the formation of volatile flavor substances in the final products (Ruan et al., 2023). The volatile flavor compounds in BA30, BA32, BA34, BA62, BA98, BA36, BA64, BA38, BA66 and BA68 were similar, which mainly included acetoin (M), 3-methyl butanol (M, D, T), trimethylamine (M, D), 2-methyl butanal (M, D), 3-methyl butanal (M, D), ethyl acetate (M, D), 2-methyl propanal and butanal. After salt curing for 48 h, the flavor substances in samples began to change significantly, and the flavor substances in dry salted Spanish mackerel were similar under 3 % salt concentration and 6 % salt concentration and were grouped into one group.

### 3.3 Microbial composition and its dynamic relationship with volatile flavor compounds

**Fig. 6:**
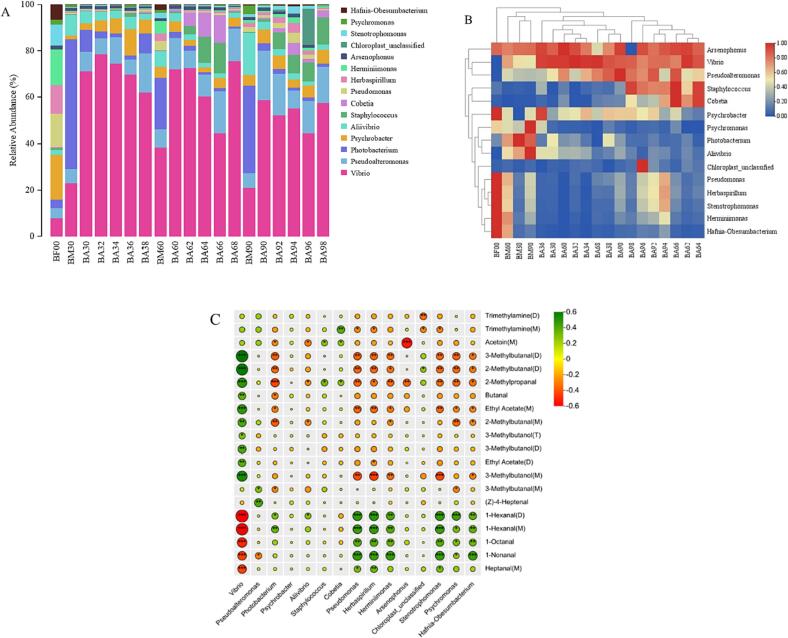
Changes in stacked bar chart at genus level (A), heat-maps (B) and correlation analysis between main volatile flavor substances and dominant bacteria (C) of dry salted Spanish mackerel under different treatment conditions. *, *p* < 0.05, **, *p* < 0.01, ***, *p* < 0.001. *p* < 0.05 was considered significant.

The microflora was analyzed and identified by 16 s high-throughput sequencing. The sequence coverage of all Spanish mackerel samples in the curing and drying process was more than 99 %, indicating that the sequencing results can truly reflect the sample information and meet the requirements of strain diversity analysis (Karoline, Vidal, Godoy, Andrea, & Rodrigues, 2023). As shown in Fig. 6A, the distribution of Top15 bacteria in different dry salted Spanish mackerel samples was analyzed at the genus level. It can be seen from Fig. 6A that the fresh samples contained the most species of bacteria and the relative content difference of each genus was small. After curing for 24 h, the contents of Vibrio and Photobacterium in the samples increased significantly, while the contents of other genera decreased gradually. With the increase of curing and drying time, the content of *vibrio* increased at first and then decreased, which reached the maximum in the samples cured at 3 % salt concentration for 2 days, but in the samples dried for 8 days at 6 % and 9 % salt concentration, the *vibrio* content reached the maximum at the same level of salt concentration. The relative content of *photobacterium* was the highest when it was pickled at 3 % salt concentration for 24 h, and at the same time, when it was cured at 6 % salt concentration, the relative content was the least. Bahrndorff et al. (2022) conducted a comparative analysis on microorganisms in traditional salted and dried fish from various countries, revealing that *Photobacterium, Psychrobacteria, Vibrio,* and *Pseudomonas* were the predominant bacterial groups present in these samples. These findings align with our own results. The relative content of the top 15 bacteria genera with abundance at the genus level was normalized and the heat map was drawn, which was enabled to compare the differences between bacteria genera under different treatment conditions more visually. Suo et al. (2023) employed the same methodology to analyze the microbial community dynamics during glutinous rice dumpling production. Each row represented the relative content of the same bacteria in different samples, and each column represented the relative content of different bacteria in the same sample. The darker the red was, the higher the relative content of the bacteria.

The contents of *Psychromonas, Pseudomonas, Herbaspirillum, Stenotrophomonas, Herminiimonas, Hafnia-obesumbacterium* in fresh samples were relatively high, and the dominant bacteria in the samples changed gradually with the change of treatment conditions and time, the contents of *Arsenophonus, Vibrio,* and *Pseudoalteromonas* were relatively high during the drying period. According to the clustering results, the salted samples with 3 % salt concentration were clustered with the post-pickling samples with high salt concentrations (6 % and 9 %), while the salted samples with 6 % and 9 % salt concentrations were clustered into the same group during the drying period, the relative contents of *Staphylococcus* and *Cobetia* in this kind of samples were higher than those of other samples. It has been reported that *Staphylococcus* and *Cobetia* are the most abundant in dry salted fish samples, and they play an important role in the ripening of dry salted fish (Xiao, Liu, Chen, Xie, & Li, 2020).

Microbial metabolism affects flavor formation in dry-cured fish through a large number of biochemical pathways. Pearson correlation coefficient was used to analyze the correlation between the main flavor components of Spanish mackerel and TOP15 bacteria in the curing and drying process. As shown in Fig. 6C, green indicated positive correlation, red indicated negative correlation, the dot represented correlation, and the larger the dot was, the greater the correlation between the two. *Vibrio* were positively correlated with 3-methyl butanol (M), ethyl acetate (M), 2-methyl propanal, 2-methyl butanal (D) and 3-methyl butanal (D), and negatively correlated with heptanal (M), 1-nonanal, 1-octanal and 1-hexanal (M, D). *Pseudomonas, Herbaspirillum, herminiimonas, stenotrophomonas, psychromonas* and *Hafnia-obesumbacterium* were positively correlated with 1-nonanal, 1-octanal, 1-hexanal (M, D), and negatively correlated with 3-methylbutanol (M), ethyl acetate (M), 2-methylpropanal, 2-methylbutanal (D) and 3-methylbutanal (D). There was a significant negative correlation between *Arsenophonus* and acetoin (M), but little correlation with other volatile flavor compounds. *Staphylococcus* exhibited a positive correlation with acetoin, thereby facilitating the rapid development of flavor and retarding lipid oxidation (Li et al., 2022, Li et al., 2022, Li et al., 2022). Furthermore, *Staphylococcus* demonstrated gradual utilization of carbohydrates, leading to their conversion into organic acids and aromatic compounds such as 2,3-butanedione, acetaldehyde, and acetoin (Liu et al, 2023). Indicating that the formation of flavor substances was closely related to the structure of the bacterial community.

## 4 Conclusions

The Dalian mackerel is used as the research object in this paper to investigate the changes in safety indices and flavor compounds during curing and drying. The TBARS value increased progressively as the salt concentration increased and the treatment period increased, demonstrating that excessive salt promotes fat oxidation. To ensure safety, the salt concentration should be lowered in order to reduce the degree of fat oxidation. The variation trend of TVB-N value and TVC value was consistent, indicating that the degree of protein degradation was mostly dependent on microbial metabolism. The TVBN value of the samples treated with 3 % salt concentration exceeded the safety limit and was therefore unfit for human consumption. GC-IMS found 61 volatile flavor compounds, including 23 aldehydes, 8 ketones, 12 alcohols, 4 esters, 4 amines, and 10 unknown components. 20 kinds of flavor compounds with ROAV ≥ 1 were selected, with aldehydes contributing the most to the overall flavor. The bacterial structure analysis of dry salted mackerel treated with HTS revealed that *Staphylococcus* and *Cobetia* were the prevalent bacteria. These findings contribute to understanding the link between bacteria and volatile taste molecules. Controlling the growth of spoilage bacteria and increasing the synthesis of positive taste compounds can improve the safety and quality of dry salted Spanish mackerel. It offers theoretical guidelines for industrial and consistent manufacturing of dry salted Spanish mackerel.

## Author contributions

All authors’ contributions as follows: Pengfei Jiang designed and directed the entire experiment, and revised the manuscript. Caiyan Jiang performed the experiments, analyzed the data and wrote the paper. Yang Liu, Wengang Jin, Kaiyue Zhu, Xiaoqing Miao and Xiuping Dong provided assistance with materials and methods. All authors approved the final version of the manuscript.

## CRediT authorship contribution statement

**Caiyan Jiang:** Writing – review & editing, Writing – original draft, Data curation. **Yang Liu:** Investigation, Data curation. **Wengang Jin:** Visualization, Validation, Resources. **Kaiyue Zhu:** Writing – review & editing, Data curation. **Xiaoqing Miao:** Investigation, Data curation. **Xiuping Dong:** Validation, Supervision. **Pengfei Jiang:** Validation, Supervision, Resources, Project administration, Funding acquisition.

## Declaration of competing interest

The authors declare that they have no known competing financial interests or personal relationships that could have appeared to influence the work reported in this paper.

## Data Availability

Data will be made available on request.
